# Santino’s growth chamber: A chamber for studies with *Aphelenchoides besseyi* on plants

**DOI:** 10.1016/j.mex.2022.101780

**Published:** 2022-07-07

**Authors:** Santino A. da Silva, Andressa C.Z. Machado

**Affiliations:** a Universidade Estadual de Londrina, PR, Brasil; b Instituto de Desenvolvimento Rural do Paraná – IAPAR/EMATER, Londrina, PR, Brasil

**Keywords:** Foliar nematodes, Plant moisture, Green stem and foliar retention syndrome, Growth chamber

## Abstract

The soybean green stem and foliar retention syndrome (GFSR) [1], caused by *Aphelenchoides besseyi*, is reported in Brazilian fields located at the States of Mato Grosso, Pará, Amapá, Tocantins, and Maranhão, which correspond to warm climates with well-defined rainfall patterns when the air humidity is high during several consecutive days. Studies showed that the infection of plants by *A. besseyi* occurs in regions with a high frequency of rains and under temperatures higher than 28 °C, when the nematode founds adequate conditions to migrate from soil to shoot parts of plants to initiate its parasitism [2]. One of the challenges was the difficulty in simulating the natural conditions for the disease development, which needs luminosity, high temperatures, and moisture. These conditions can be reproduced using modern growth chambers, but these equipment are onerous and scarce in most Brazilian research centers. So, for the studies with *A. besseyi* and different plant hosts, it is difficult to reproduce the environmental conditions similar to those found in the field where this nematode is reported, especially under the operational and economic points of view. Considering these environmental conditions and the necessity in conducting studies under controlled environments with this pathossystem aiming a detailed investigation about the symptoms and the nematode parasitism, but also to isolate the effects due to exclusively the nematode parasitism instead of other effects that occur under field conditions, especially in crops like soybean, common bean, and cotton [1], [2], [3], [4], the objective of this project was to develop a growth chamber for the cultivation of these plants under controlled environmental, simulating the necessary conditions for the GFSR development. For this, we used an environmental chamber [5] as the base to our project, where we could find the important aspects that need to be adjusted to suit our purpose.

• We developed a plant growth chamber to be used under greenhouse conditions for the studies with *Aphelenchoides besseyi* and different host plants.
• The soybean green stem and foliar retention syndrome (GFSR) needs specific environmental conditions of humidity and temperature for the development of the characteristic symptoms and for the nematode multiplication in the parasitized plants.
• This method simulates adequately the environmental conditions found in the field, since the chamber is installed inside a greenhouse, assuring the reliable observation of the plant behavior in relation to the pathogen and allowing the conduction of experiments of this nature in regions different from those where the disease naturally occurs.

## Graphical abstract

**Figure:**
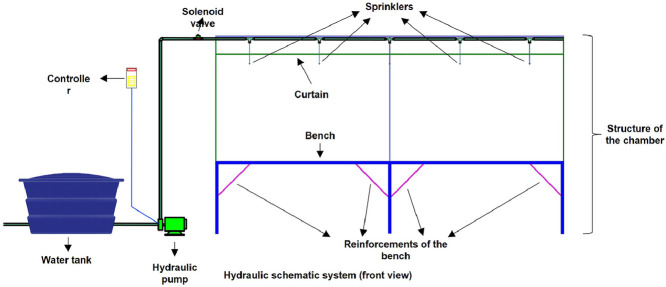

**SPECIFICATIONS TABLE**

**Table:** 

Subject Area:	Agricultural and Biological Sciences
More specific subject area:	Phytopathology (Nematology)
Method name:	Growth Chamber for the study of the green stem and foliar retention syndrome caused by *Aphelenchoides besseyi*
Name and reference of original method:	Environmental Chamber for Plant Growth Analysis. Griggs, S.H; Bartley, W.E; Rule, A.O. United States Patent. Patent number 5341,595, Aug. 30, 1994.
Resource availability:	None

## Design of the Santino's growth chamber for GSFR studies

**Fig. 1:**
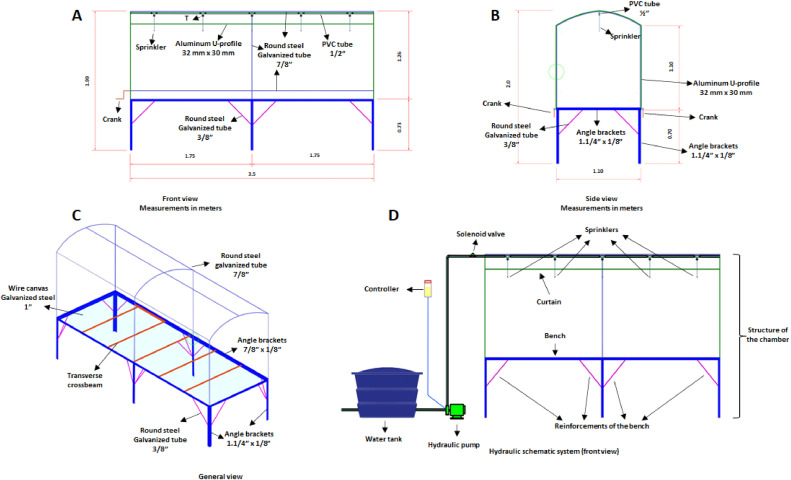
Schematic structure of the Santino's growth chamber.

For the confection of the growth chamber (Fig. 1A), we used drilling screws for fixation of the pieces; metallic plates of 2 mm bent in L (5 cm of length each side and 2 cm of width), used to fix the tubular bases of the chamber to the bench; round steel galvanized tube 7/8, used for the production of the tubular structure of the growth chamber, and cranks (made by bending the steel tubes) for opening the curtains (diffuser plastic), movable sides; 12 (zigzag), galvanized steel wire, to fix and fasten the plastic to the structure; aluminum U-profile fixed to the tubular structure, used to fit the plastic and to fasten with the zigzag wire; 5 Super Fogger x 4 m sprinklers, for medium pressure (flow rate of 20.8 L/h), ½ inch PVC pipe (according to the distance in the installation place), PVC tank (100 L), 5 T with thread to fix the couplers for the sprinklers, bends and PVC connections, according to the place of installation and to the need in make angles to install the hydraulic system.

The galvanized steel tubes were shaped and bent to form the pieces that were then fixed to form the structural frame of the growth chamber. The right foot of the structure was 1.10 m high from the bench or 1.8 m from the ground, the central part of the structure (in the shortest extension of the bench), in the shape of a vault with 35° curves, was 1.26 m high from the bench or 1.96 m from the ground (Fig. 1B).

A bench (Fig. 1C) was built using angle brackets with the dimensions of 1.1/4 × 1/18 inches, in galvanized steel, measuring 1.10 m wide, 0.73 m high, and 3.5 m long, with the angle brackets that form the base of the bench welded with the edges facing upwards. Every 0.583 m, a transverse crossbeam was welded, with the same thickness, to support and maintain the structure (Fig. 1C). Angle brackets were welded at the ends of the rectangle and the center of the rectangle on the two sides, forming the feet of the bench. Wire canvas 12 (2.65 mm thick), in galvanized steel, with a 1-inch opening, was welded to the free span of the assembled structure, thus forming the bench on which the growth chamber structure was mounted.

## Structure details

As indicated in Fig. 1, we can see the front view of the structure, dimensions, materials used, position of the nebulizers and scheme of the chamber side curtains in A; in B, the side view of the end of the structure, materials, height of the rectangular part and vault of the chamber, arch with fixed plastic (without curtain), position of the hydraulic line for the nebulization system (the green circle indicates the support to leave the curtain semi-open), in C, the three-dimensional top view of the structure, position, organization of the metallic structures of the upper chamber, view of the bench structure and arrangement of the galvanized screen, which serves as basis for the arrangement of pots with plants, and in D, the functional diagram of the chamber, water tank for the system, controller to regulate the interval and duration of water spray inside the chamber, hydraulic pump to pressurize the system and ensure volume and size of water droplets compatible with the necessary nebulization, solenoid valve that controls the release of nebulization in each chamber (when in a system composed of more than one chamber), diagram of the physical structure of the chamber, from a front view, indicating the position of the necessary and adequate components of the chamber, position of sprinklers and arches, sides and tops, in addition to the bases and reinforcements of the bench.

A straight galvanized steel tube, measuring 3.5 m long, was fixed in the center of the upper part of the vault in the direction parallel to the longest extension of the bench to support the roof structure and to fix the misting system. To fix the plastic on the roof, front sides, and movable side curtains, U-profiles were fixed, being 1.10 m high on the right feet lateral (in the parallel part of the bench), 1.10 m at the top of the arch (in the base of the vault), front view of the table, and on the external sides, where the plastic of the roof and later the plastic of the curtains and front sides were fixed. C-shaped wires were installed at the right feet at the ends to hold the raised curtains in two positions, 40 and 90 cm high from the bench.

## Misting system

For misting, a system (Fig. 1D) was installed consisting of a 100-liter water tank, a 0.3 HP motor-pump set, a filter to prevent clogging of the sprinklers, piping with ½-inch PVC pipe bars, with the piping fixed in the center of the vault of the growth chamber. Five sprinklers were installed, 0.75 m apart from each other and 0.25 m away from the ends of the growth chamber, connected to the water distribution line by threaded T, where the couplings for the sprinklers that were at a height of 1.05 m from the bench were coupled.

**Fig. 2:**
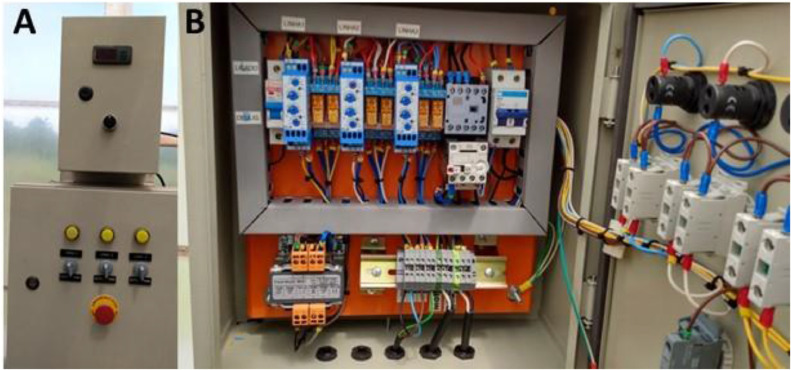
Electric controller system of the Santino's growth chamber.

After the construction of the structure, a system was installed to control the schedules and misting time, with a control powered by a transformer with 220 V current input and 24 VAC output, control by 24 VAC relays, cyclic timer, promoter contactor, thermal relay, 20 A circuit breaker, output terminals, and control buttons (Fig. 2).

In Fig. 2, the misting control system is specified as: A) above - water temperature controller used for nebulization in the chamber, programmed to keep the water at a minimum of 25 °C, control panel and button to turn the system on and off; below - front view of the nebulization system controller, each button corresponds to the activation of the system in a chamber, automatic, manual and on-off functions, emergency button in case of system failure; B) view of the system's control components, consisting of circuit breakers, analog cyclic timers, activation coils, contactor, phase failure relay, source for current conversion from 220 V to 12 V, terminals for electrical current distribution.

## Preliminary tests and adjustments

**Fig. 3:**
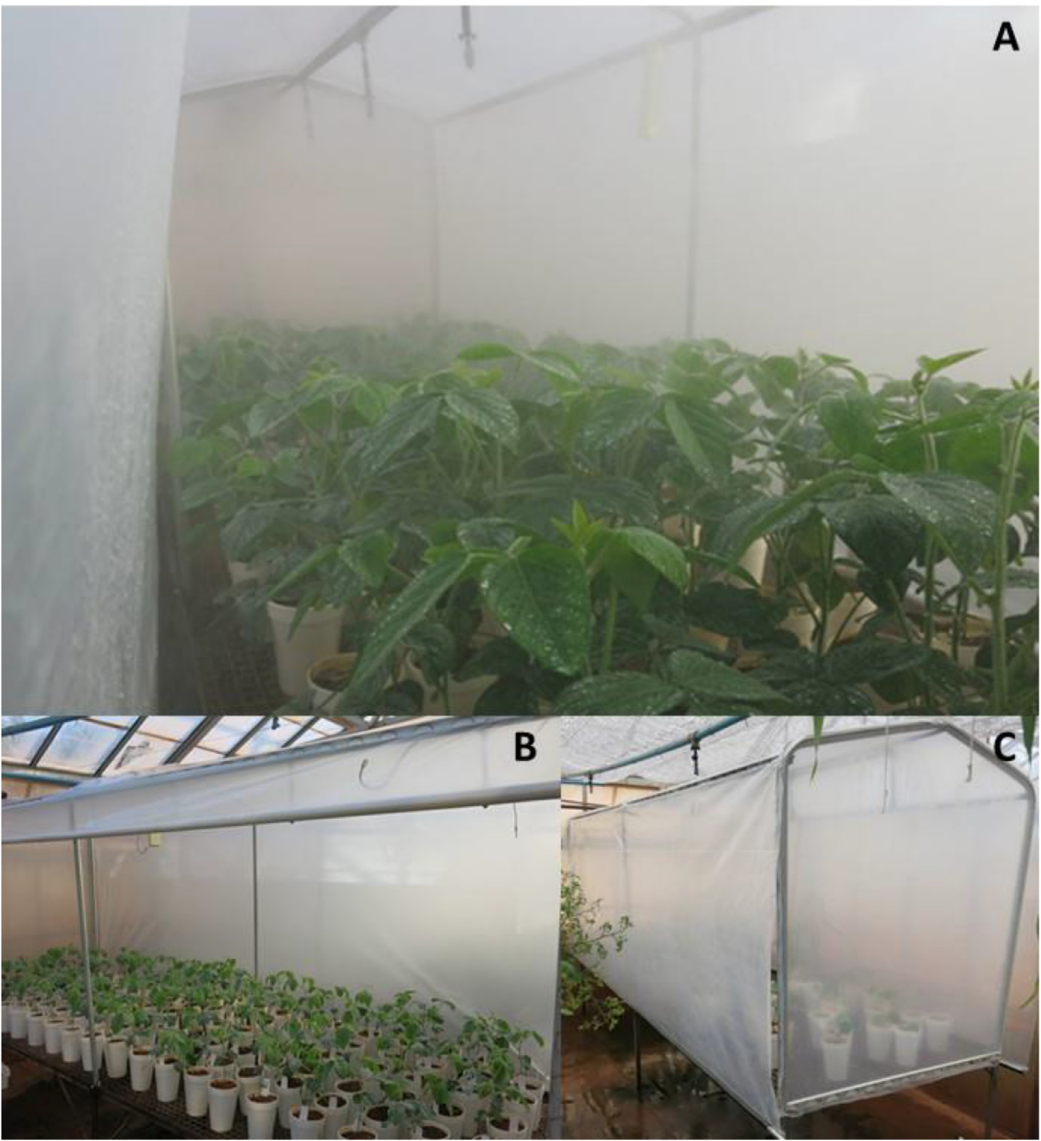
Functioning of the Santino's growth chamber.

After preliminary tests, it was verified that the best configuration is the irrigation cycle type (on 24 h), being programmed to have from 5 to 30 s of misting every 15 min (the interval is always fixed, with the possibility to alter the time of irrigation) (Fig. 3A).

At the beginning of the experiment, until about 15 days after germination or transplanting, a misting time of 5 s per start was used and, as the plants grew, this time was adjusted so that there was always humidity on the leaves.

Table 1.

**Table 1:** Adjustments related to cultivated crops in Santino´s GC regarding humidity conditions and intervals between misting.

Crop	Plant age at inoculation	Initial population density of inoculum	Interval between misting	Period time of misting
*Phaseolus vulgaris*	4 - 9	300 - 500	15 - 30 min	5 - 30 s
*Glycine max*	7 - 12	300 - 500	20 - 30 min	5 - 30 s
*Gossypium hirsutum*	15 - 20	300 - 500	10 - 30 min	5 - 30 s

The time of germination for each crop species, the crop cycle and the development of plants were considered to the adjustments done in the misting chamber. Due to the intrinsic characteristics of each crop species, as hairy and leaf area surface, which interfere in the period in which the water microdroplets dry, these adjustments were necessary. In the beginning of the crop cycle, we can adopt higher intervals between misting and, according with the development of plants, this interval must be reduced, in order to maintain the homogeneity in the humidity conditions for each crop species.

Three growth chambers were built as a way to maintain the independence in the programming between them, a solenoid valve was installed between the hydraulic master line and the misting system of each chamber, in a way to allow that experiments could be conducted simultaneously but simulating different conditions, a controller was installed for each chamber (Fig. 2).

To ensure that there was no fluctuation in the temperature of the water used in the nebulization, a resistance set was installed inside the water tank that feeds the system, so whenever the temperature dropped to 25 °C it was activated, preventing lower temperature of the water from misting in the growth chamber than the programmed, since low temperatures could be a limiting factor to nematode development.

In the first tests, critical points were observed in the process, and adjustments were done aiming the efficacy of the system, according to indicated in Fig. 3, in which we can see: A) mist and moisture in the leaf surface of soybean plants; B) side view of the chamber with one of the curtains open, arrangement of plants inside the chamber, showing the large volume of plants that can be cultivated at the same time; C) view of the chamber with the side curtains closed and the fixed part of the chamber arc (operating mode of the chamber when the system is on).

## Santino's growth chamber validation

Using Santino's GC we conducted experiments with soybean (Fig. 3B, C), cotton, common bean, and tobacco. For this, a pure population of *A. besseyi* used as inoculum was multiplied in *Fusarium* cultures on potato-dextrose-agar medium [1]. Nematodes (300 per plant) (initial population = IP) were inoculated on common bean, soybean, and cotton plants after 6, 10, and 20 days after sowing, by pipetting a suspension containing the nematodes into the soil, near the plant stem.

Temperature and humidity inside and outside to the growth chamber were annotated daily. The experiments were evaluated at 30 days after the inoculation (DAI), verifying the development of the characteristic symptoms of GSFR. All inoculated plants showed these symptoms, confirming the viability of the growth chamber conditions to disease development.

**Fig. 4:**
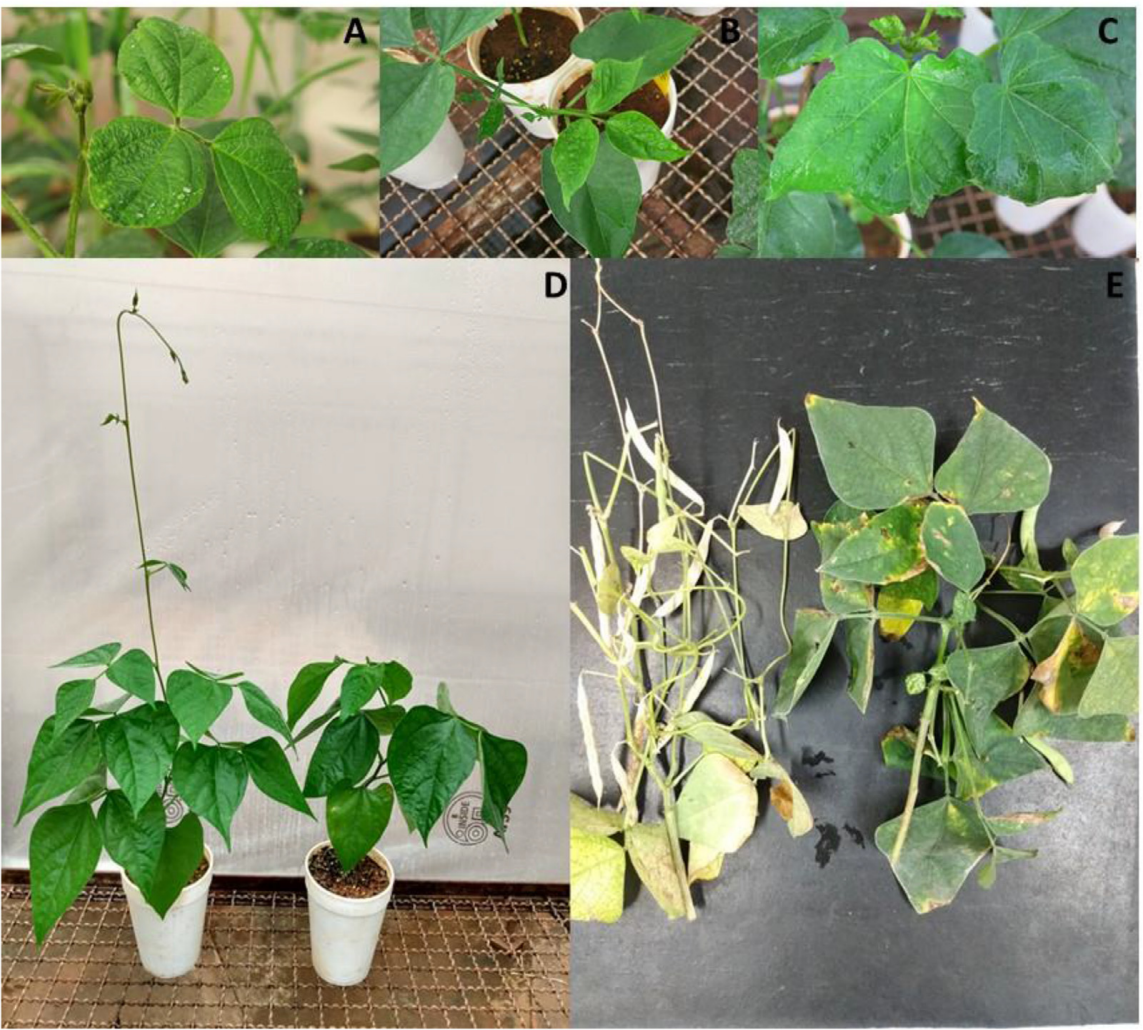
Symptoms of the green stem and foliar retention syndrome in soybean, common bean, and cotton plants, obtained in the Santino's growth chamber.

Experiments were also performed on soybean and common bean plants with inoculated and non-inoculated plants in the same chamber, with the aim of verifying whether the symptoms observed are due to nematode parasitism or exclusively to the environmental conditions inside the chamber. The chamber was divided into two halves by a plastic curtain, fixed on the right central feet of the chamber, at a height of 85 cm from the bench. Until 60 DAI, there was no contamination of the non-inoculated plants, which were healthy with normal development, while the inoculated plants showed all the characteristic symptoms of the disease (Fig. 4).

After the adjustments and the studies conducted to validate the growth chamber, the symptoms characteristic from the GSFR were frequently observed, as indicated in Fig. 4:A) leaf wrinkling in soybean; B) “amachamiento” in common bean leaves; C) wrinkling in cotton leaves; D) Common bean plants non-inoculated (left) and inoculated (right) with *Aphelenchoides besseyi*, conducted at the same time in the chamber, but physically separated by a plastic curtain, showing the symptoms of the syndrome at 30 days after inoculation; D) Common bean inoculated (left) and non-inoculated (right) at 60 days after inoculation.

At 35 DAI, five soybean plants inoculated with *A. besseyi* were evaluated through the optimized Baermann funnel for nematode extraction from soil, through the blender-sieving methodology for nematode extraction from roots, and through the blender-sieving + optimized Baermann funnel for nematode extraction from shoot parts [6]. The number of nematodes extracted from soil, root, and shoot was added to totalize the final population (FP) of the nematode, which was used to calculate the reproduction factor (RF = IP/FP) [7]. Based on the RF values, we concluded that *A. besseyi* multiplied in the inoculated plants (Table 2), confirming the adequate conditions inside the growth chamber to nematode development and multiplication.

**Table 2:** Number of nematodes extracted from soybean plants inoculated with 300 *Aphelenchoides besseyi* at 30 days after inoculation (DAI).

NNS	NNR	NNAT	FP	RF
191	213	341	745	2.48

Values are means of five replicates. NNS = number of nematodes extracted from soil; NNR = number of nematodes extracted from roots; NNAT = number of nematodes extracted from shoots; FP = final population (NNS+NNR+NNAT); FR= reproduction factor.

## Conclusions

Results allowed us to conclude that the growth chamber is adequate to conducting experiments with soybean, common bean, cotton, and tobacco inoculated with *A. besseyi.* It is important to note that, under the humidity and temperature conditions obtained in the growth chamber, only the inoculated plants showed the characteristics symptoms of the GSFR syndrome, reinforcing that the symptoms are not induced by the environmental conditions but by the nematode parasitism. Therefore, in the same chamber, divided in two parts with the plastic curtain, we can study inoculated and non-inoculated plants under the same environmental condition, a primordial situation for experimentation.

Therefore, the controlled cultivation system installed inside a greenhouse (Santino´s GC) guarantees all the adequate conditions to carry out experiments aiming to study the GSFR syndrome with low cost and being easy fabrication and maintenance. We also concluded that the environmental conditions, i.e., high humidity and temperature close to 28 °C [2] obtained inside the growth chamber, are determinant to the visualization of the symptoms associated with the GSFR, the multiplication of *A. besseyi* in the plants, and the disease development.

## Declaration of Competing Interest

The authors declare that they have no known competing financial interests or personal relationships that could have appeared to influence the work reported in this paper.

## Data Availability

Data will be made available on request.
